# Short-term effective treatment of CNS metastasis of sarcomatoid renal cell carcinoma with temozolomide and pegylated liposomal doxorubicin: A case report

**DOI:** 10.1186/1757-1626-1-210

**Published:** 2008-10-03

**Authors:** Dagmar Beier, Gerhard Schuierer, Christoph P Beier, Ulrich Bogdahn

**Affiliations:** 1 Department of Neurology, Medical School, University of Regensburg and District Medical Center Regensburg, Universitätsstrasse 84, 93053 Regensburg, Germany; 2 Institute of Neuroradiology, District Medical Center Regensburg, Universitätsstrasse 84, 93053 Regensburg, Germany

## Abstract

Sarcomatoid renal cell carcinoma represents high-grade transformation of different subtypes of renal cell carcinoma and is associated with a dismal prognosis and high resistance to chemotherapy. We report on the course of disease of a 63 years old patient undergoing a nearly complete remission of multiple intracranial and spinal metastatic lesions of a sarcomatoid renal cell carcinoma by a combined chemotherapy with temozolomide and pegylated liposomal doxorubicin.

## Case presentation

In December 2004, a 63-year-old female, Caucasian patient presented in our hospital with progressive paraplegia due to multiple CNS metastasis of renal cell carcinoma (RCC). Already in January 2003, she had noticed a slowly progressive numbness of her right forearm and belt-shaped pain of her chest due to metastatic disease at the level of T1/2 causing compression of the spinal cord. Both, the RCC and the metastatic lesion were completely removed in February 2003. Pathological analysis of the renal cell tumor revealed a clear cell RCC, the intraspinal metastasis was characterized by mesenchymal appearance with epithelial components and areas with spindle cells. Based on the histological findings, the National Reference Center of Neuropathology made the diagnosis of an undifferentiated sarcoma – most likely a sarcomatoid metastasis of the clear cell RCC.

Spinal surgery was followed by local radiotherapy (cervical and upper thoracic spine, 50 Gy; primary tumor site: 59.4 Gy) and chemotherapy with temozolomide (three cycles, 1600 mg per cycle). After one year, MRI revealed multiple asymptomatic relapses affecting the clivus, C6, and T11-S1. Another local radiotherapy (Clivus-C6 and T11-S2, 50 Gy) was administered until July 2004. In July 2004, the patient noted for the first time progressive prickling dysesthesias due to a new recurrence affecting T5-10. The patient then started palliative chemotherapy with thalidomide (200 mg orally every day).

**Figure 1:**
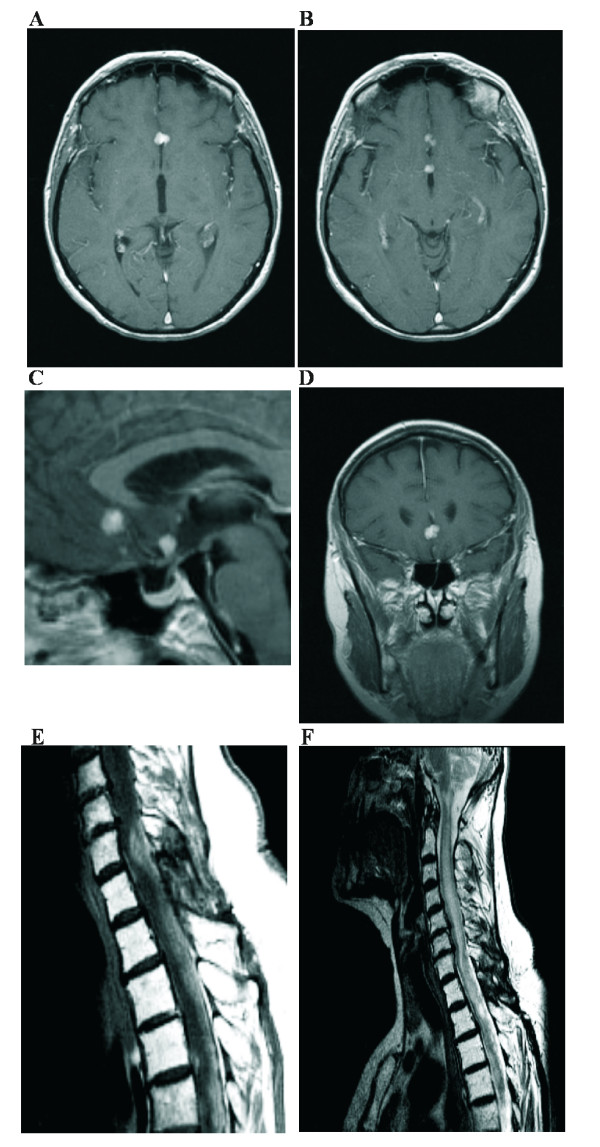
**December 2004:** Contrast-enhanced axial (A, B), sagittal (C), and coronal (D) T1-weighted MR-images of the brain and sagittal T1- and T2-weighted MR-images (E, F) of the spine showing multiple intracranial metastatic nodules and extensive spinal involvement.

**Figure 2:**
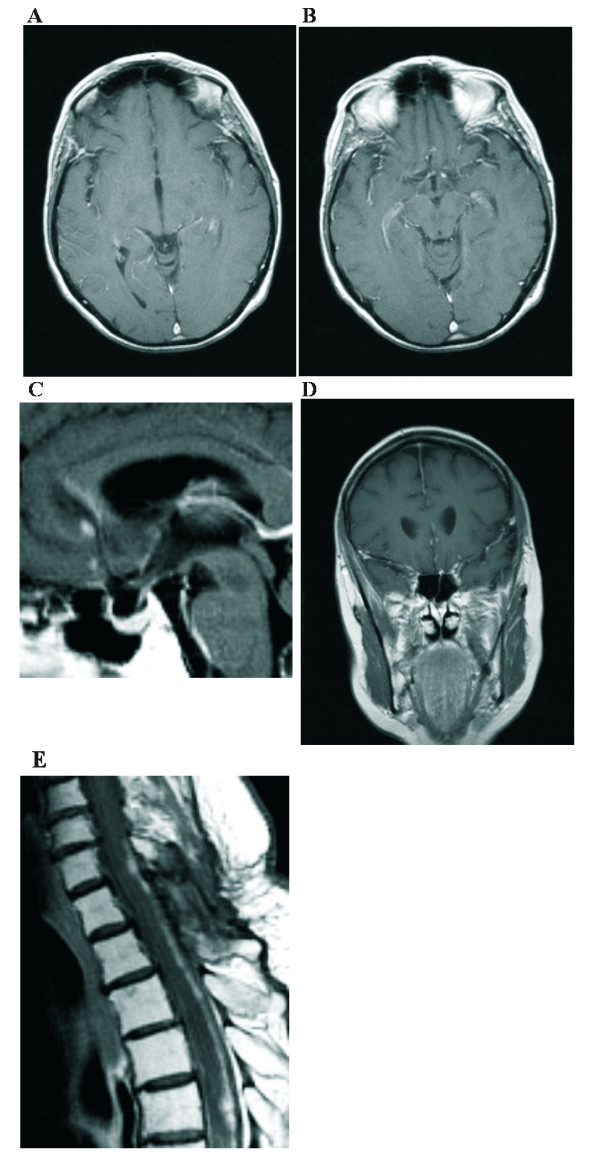
**January 2005:** Contrast-enhanced axial (A, B), sagittal (C), and coronal (D) T1-weighted MR-images of the brain and sagittal T1-weighted MR-image (E) of the spine demonstrating significant regression of the tumor.

Until December 2004, the patient further deteriorated and was finally unable to stand when presenting in our hospital. MRI showed a marked progression of all metastatic lesions in the CNS (Figure 1). We initiated chemotherapy with temozolomide orally (200 mg/m^2^) on five consecutive days every four weeks and pegylated liposomal doxorubicin intravenously (20 mg/m^2 ^every two weeks). In addition, we treated the patient with methylprednisolone, glycerine, and thalidomide. The patient's clinical condition improved rapidly and four weeks later, she was able to climb four storeys with her walking frame. In January 2005, MRI showed a nearly complete remission of the intracranial metastases and a partial remission of the spinal lumps (Figure 2). The patient received the first cycle of chemotherapy regularly. Then, prolonged myelosuppression required dose reduction and finally the discontinuation of the chemotherapy before finishing the second cycle. In addition, the port for chemotherapy got infected and multiple septic abscesses occurred. After discontinuation of chemotherapy in March 2005 the symptoms reocurred due to regrowth of the metastases. As signs of inflammation still reoccurred intermittently, we were not able to restart chemotherapy. The patient was discharged and died in June 2005 due to progressive disease.

## Discussion

Sarcomatoid RCC is not a distinct entity but represents a common histological phenotype after high-grade transformation of different subtypes of RCC [1]. It is associated with a dismal prognosis with tumors metastasizing earlier [1,2]. RCC are usually resistant to classical chemotherapeutic agents but there are several small case studies reporting on partial remissions of sarcomatoid RCC after doxorubicin-based chemotherapeutic protocols [3-7]. The rationale for combining temozolomide and pegylated liposomal doxorubicin was based on their anti-tumor activity, their good penetration of the blood-brain barrier, and no significant overlapping toxicities. The combined therapy usually is well tolerated [8,9] and severe adverse events as seen in our patient are uncommon. To our knowledge, this is the first report on the effective treatment of multiple CNS metastasis of a sarcomatoid RCC with temozolomide and pegylated liposomal doxorubicin suggesting that the combination is treatment option for recurrent CNS metastasis of sarcomatoid RCC.

## Abbreviations

RCC: renal cell carcinoma; CNS: Central nervous system; MRI: magnetic resonance imaging; C1: cervical vertebra 1; T1: thoracic vertebra 1; S1: sacral vertebra 1.

## Competing interests

Christoph P. Beier has received reimbursements by Schering-Plough, Ulrich Bogdahn has received honoraria by Schering-Plough. Dagmar Beier and Gerhard Schuierer do not have competing interests.

## Authors' contributions

DB and UB were the treating physicians of the patient DB, CPB, and UB interpreted the data and wrote the manuscript. GS provided the MRIs. All authors read and approved the manuscript.

## Consent

Because the patient has deceased, written informed consent was obtained from the patient's husband for publication of this case report and accompanying images. A copy of the written consent is available for review by the Editor-in-Chief of this journal.
